# A Refined Human Linear B Cell Epitope Map of Outer Surface Protein C (OspC) From the Lyme Disease Spirochete, *Borrelia Burgdorferi*

**DOI:** 10.20411/pai.v10i1.756

**Published:** 2025-02-14

**Authors:** Grace Freeman-Gallant, Kathleen McCarthy, Jennifer Yates, Karen Kulas, Michael J. Rudolph, David J. Vance, Nicholas J. Mantis

**Affiliations:** 1 Division of Infectious Diseases, Wadsworth Center, New York State Department of Health, Albany, New York; 2 Department of Biomedical Sciences, University at Albany, Albany, New York; 3 New York Structural Biology Center, New York, New York

**Keywords:** Lyme disease, antibody, epitope, vaccine, *Borrelia burgdorferi*, *Borreliella*, human, peptides

## Abstract

**Background:**

A detailed understanding of the human antibody response to outer surface protein C (OspC) of *Borrelia burgdorferi* has important implications for Lyme disease diagnostics and vaccines.

**Methods:**

In this report, 13 peptides encompassing 8 reported OspC linear B-cell epitopes from OspC types A, B, and K, including the largely conserved C-terminus (residues 193–210), were evaluated by multiplex immunoassay (MIA) for IgG reactivity with ~700 human serum samples confirmed positive in a 2-tiered Lyme disease diagnostic assay (Bb^+^) and ~160 post-treatment Lyme disease (PTLD) serum samples. The *vmp*-like sequence E (VlsE) C6-17 peptide was included as a positive control.

**Results:**

Serum IgG from Bb^+^ samples were reactive with 10 of the 13 OspC-derived peptides tested, with the C-terminal peptide (residues 193–210) being the most reactive. Spearman's rank correlation matrices and hierarchical clustering revealed a strong correlation between 193–210 and VlsE C6-17 peptide reactivity but little demonstrable association between 193–210 and the other OspC peptides or recombinant OspC. OspC peptide reactivities (excluding 193–210) were strongly correlated with each other and were disproportionately influenced by a subset of pan-reactive samples. In the PTLD sample set, none of the OspC-derived peptides were significantly reactive over baseline, even though VlsE C6-17 peptide reactivity remained.

**Conclusions:**

The asynchronous and potentially short-lived serologic response to OspC-derived peptides reveals the complexity of B-cell responses to *B. burgdorferi* lipoproteins and confounds interpretation of antibody profiles for Lyme disease diagnostics.

## INTRODUCTION

Lyme disease, also known as Lyme borreliosis, is the most common vector-borne infection in the United States, with an estimated 450,000 cases per year [1]. The primary etiologic agent of Lyme disease is the spirochetal bacterium, *Borrelia burgdorferi* sensu stricto^1^. In North America, *B. burgdorferi* is transmitted to humans by black-legged ticks, *Ixodes scapularis* and *Ixodes pacificus*. The spirochete proliferates at the site of the tick bite, typically resulting in an expanding skin lesion commonly referred to as erythema migrans [2–4]. In the absence of antibiotic intervention, *B. burgdorferi* can disseminate to peripheral tissues, organs, joints, and the central nervous system, potentially resulting in complications including neuroborreliosis, carditis, and/or Lyme arthritis weeks, months or even years later [2, 5]. Moreover, a small fraction of patients with Lyme disease who receive a full regimen of antibiotics will report persistent health issues (eg, fatigue, cognitive issues, musculoskeletal pain), a difficult to define syndrome termed post-treatment Lyme disease (PTLD) [6–9]. Developing rapid and accurate diagnostic tests capable of detecting and discriminating between LD, PTLD syndrome and *B. burgdorferi* reinfection are much needed [2, 10, 11].

The humoral response to *B. burgdorferi* is robust, resulting in detectable spirochete-specific B cells and serum IgM and IgG days and weeks following an infectious tick bite [12–15]. In patients with Lyme disease, antibodies are primarily directed against *B. burgdorferi*'s outer surface lipoproteins [16]. Among these, outer surface protein C (OspC; also known as BB_B19, P23 and P25) stands out. OspC is a ~21 kDa helical homodimeric lipoprotein expressed by *B. burgdorferi* during tick transmission and in the early stages of infection [17]. OspC is implicated in facilitating spirochete egress from the tick during the course of a blood meal, enabling survival in the early stages of mammalian skin infection, and modulating transmigration across vascular walls [17–24]. The highly immunogenic nature of OspC has lent itself to applications in Lyme disease serology-based diagnostics, including widely used commercial tests [25, 26]. For example, the peptide derived from the largely conserved C-terminus of OspC (peptide “C10”) is a component of the FDA-approved diagnostic test *Borrelia* VlsE1/pepC10 from Zeus Scientific [27–31]. In addition to their diagnostic utility, linear B-cell epitopes from different OspC types are a component of a widely used canine Lyme disease vaccine and are being considered for human use (see review article on this topic [32]).

While multiple linear human B-cell epitopes on OspC have been reported over the past 3 decades, differences in serologic assays, detection methodologies, and sample sizes from varying Lyme disease cohorts make it difficult to draw conclusions about relative reactivities of one peptide over another [28, 31–41]. This is problematic because determining the relationships between OspC-derived peptides and total OspC antibodies has practical implications for interpreting serologic as-says. Moreover, there are >26 allelic variants or types of OspC reported in the United States, with amino acid sequence identities that range from 60% to 90% [42, 43]. As different OspC types are associated with varying degrees of virulence and invasiveness, tracking epitope-specific responses to those particular types is of paramount importance [44, 45]. In New York state, for example, OspC types A, B, and K, which are associated with more invasive disease, represent ~70% of all isolates [35, 45, 46]. With that in mind, we sought to validate human antibody reactivities to reported linear epitopes associated with OspC types A, B, and K to enable broad comparability of epitope usage across *B. burgdorferi* for diagnostic and vaccine development purposes.

## METHODS

### Chemicals and Biological Reagents

Chemicals and reagents were obtained from Thermo Fisher Scientific, unless noted otherwise. Buffers were prepared by the Wadsworth Center's Cell and Tissue Culture core facility.

### Recombinant OspC and OspC-derived Peptides

Recombinant *B. burgdorferi* OspC_A_ (residues 38 to 201; PDB ID 1GGQ; UniProt ID Q07337) [47], OspC_B_ (residues 38 to 202; *B. burgdorferi* strain ZS7; PDB ID 7UJ2) [48] and OspC_K_ (residues 38 to 202; *B. burgdorferi* strain 297; PDB ID 7UJ6) [49] were expressed in *E. coli* strain BL21 (DE3) and purified by nickel-affinity and size-exclusion chromatography, as described [50]. Recombinant OspC_A_ with the C10 sequence (residues 201–210; PVVAESPKKP; “OspC_A_+C10”) was expressed and purified as above. Linear epitope prediction was done using Discotope 2.0 [51]. OspC peptides (>80% purity) as described in Table 1 were synthesized by Genemed Synthesis with a C-terminal GGGSK-biotin extension. We also synthesized the *vmp*-like sequence E (VlsE)-derived C6 B31–17 peptide (MKKDDQIAAAIALRGMA) with the GGGSK-biotin linker [52].

**Table 1.** OspC and VlsE-derived Peptides Used in This Study

#^a^	Residues	OspC type sequence (A, B, K)^b^	IEDB ID^c^	References
**1**	9–33	ILMTLFLFISCNNSGKDGNTSANSA	181204 745120	[79, 28, 39, 80]
**2**	40–55	A/B-PNLTEISKKITDSNAV K-PNLTEISKKITESNAV	559957	[39]
**3**	71–86	A-EIAAKAIGKKIHQNNG	12383	[81]
**4**	132–146 132–146 133–147	A-ETFTNKLKEKHTDLG B-EEFSTKLKDNHAQLG K-EDFTKKLEGEHAQLG	63756, 745097 14380, 18025	[36]
**5**	155–169	A-AKEAILKTNGTKTKG	558968 181187	[28, 39]
**6**	177–186 178–187 178–187	A-FESVEVLSKA B-SGSLESLSKA K-FKAVENLAKA	NA	[82]
**7**	183–190^d^	A/B-LSKAAKEM	560173	[39]
**8**	193–210	A-NSVKELTSPVVAESPKKP	745122	[26, 39, 62, 80]
**C6**	VlsE C6-17	MKKDDQIAAAIALRGMA	41843	[52]

**Figure 1.**
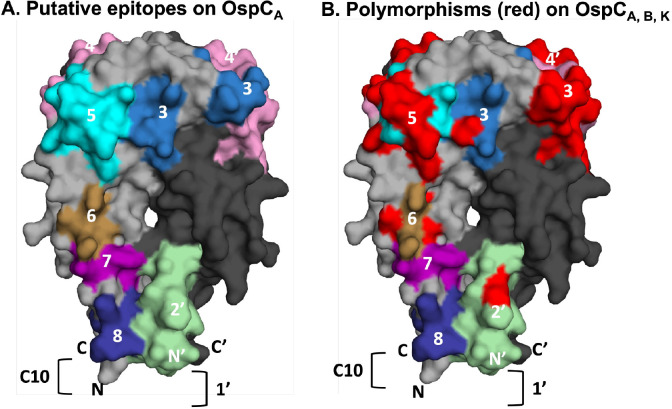
**Relative location of linear epitopes on homodimeric OspC_A_.** (A) Surface representation of homodimeric OspC_A_ (residues 38–201; PDB 1GGQ] with one monomer (OspC) colored gray and the other, denoted with an apostrophe (OspC'), colored in charcoal. The linear epitopes examined in this study are coded and numbered according to Table 1. Epitope 1 is represented as a bracket (]) on OspC', as its corresponding residues were truncated in the recombinant version of OspC_A_ used for X-ray crystal structure analysis. Similarly, only residues 193–201 of epitope 8 (residues 193–210) are shown because the C-terminus of OspC_A_ was truncated at position 201. Overlap between epitopes 6 and 7 are colored as epitope 6. (B) The same image as Panel A except that amino acid differences between OspC types A, B, and K within the 8 epitopes are colored red to illustrate degree of polymorphisms.

a, Numbers correspond to peptides highlighted in Figure 1, except C6;

b, The capital letters A, B, or K preceding the peptides indicate their OspC type sequence.

c, Immune Epitope Database (IEDB.org) identifier.

d, Residues 184–191 in Type B.

### Commercial and Clinical Lyme Disease Serum Samples

Commercial Lyme disease seronegative (Lot 10500586) and seropositive (Lot 10510438) pooled samples were used strictly as intra-assay and bead-coupling confirmation controls throughout this study (ACCURUN products 810 and 130, respectively; SeraCare). The Lyme disease seronegative samples (referred to as “controls” throughout the manuscript) consist of a commercial panel of 81 serum samples collected in 2017–2018 (Access Biologicals). Five of the 81 serum samples were classified as extreme outliers for VlsE C6-17 reactivity by interquartile range (falling outside Q3 + (3^*^IQR)) calculated using Microsoft Excel and therefore removed from the control panel. In the end, a total of 76 control samples were used as comparators with the diagnostic sample set, and 75 samples were used as comparators for the PTLD sample set as a result of sample availability.

*B. burgdorferi* seropositive (Bb^+^) serum samples (n = 696) were obtained from the Wadsworth Center's Diagnostic Immunology Laboratory. The archived samples were previously subjected to 2-tiered testing consisting of (Tier 1) a C6 peptide screen (Immunetics; C6 Lyme ELISA) or Enzyme Linked Fluorescent Assay (ELFA; BioMerieux, VIDAS Lyme IgG II and Lyme IgM II) followed by (Tier 2) IgM and IgG detection by Western blot (MarDX; Trinity Biotech). *B. burgdorferi*-specific IgM reactivity was defined as ≥2 positive bands, with IgG reactivity defined as ≥5 positive bands. Serum samples were aliquoted, de-identified, and classified as IgM-positive/IgG-negative, IgM-positive/IgG-positive, or IgM-negative/IgG-positive, based on the Western blot results. Post-treatment Lyme disease (PTLD) serum samples (n=158) were kindly provided by the Lyme Disease Biobank at Nuvance Health^®^ (Danbury, CT). PTLD was defined as described by Aucott [8].

### OspC and OspC Peptide Immunoassays by Multiplexed Microsphere Immunoassays and Enzyme-linked Immunosorbent Assay

Recombinant OspC_A_, OspC_B_, or OspC_K_ (5 μg) were coupled to Magplex-C microspheres (1 x 10^6^) using sulfo-NHS (N-hydroxysulfosuccinimide) and EDC [1-ethyl-3-(3-dimethylaminopropyl) carbodiimide hydrochloride], as recommended by the manufacturer (Luminex Corp.). Coupled beads were diluted in xMap AbC Wash Buffer to a concentration of 5 x 10^6^ beads/mL. Biotin-labeled peptides were complexed to Magplex-avidin microspheres following protocols provided by the manufacturer (Luminex Corp.). Microspheres were resuspended in 250 μL of assay buffer (1X PBS, 2% BSA, pH 7.4) then subjected to vortexing and sonication. A total of 1 x 10^6^ beads in assay buffer was mixed with biotin-conjugated peptides (~5 μg) and incubated for 30 minutes at room temperature. The microsphere suspensions were then washed 3 times using wash buffer (1X PBS, 2% BSA, 0.02% Tween-20, 0.05% sodium azide, pH 7.4) and a magnetic separator, resuspended in 500 μL of assay buffer, and stored at 4°C until use. Successful coupling of OspC and peptides to the Magplex-C microspheres was confirmed by reactivity with immune serum and/or monoclonal antibodies.

For experimental use, assay buffer was used to dilute bead stocks (1:50) and human serum (1:100). The bead dilution (50 μL) and diluted serum (50 μL) were combined in black, clear-bottomed, non-binding, chimney 96-well plates (Greiner Bio-One) and allowed to incubate for 60 minutes in a tabletop shaker (600 rpm) at room temperature. Plates were washed 3 times using a magnetic separator and wash buffer. Secondary antibody goat anti-Human IgG Fc, eBioscience (Invitrogen) was diluted 1:500 in assay buffer and added (100 μL) to each well. The secondary antibody was allowed to incubate for 30 minutes in a tabletop shaker (600 rpm) at room temperature. Plates were washed as stated above and beads were resuspended in 100 μL of wash buffer. Samples were analyzed using a FlexMap 3D (Luminex Corp). To evaluate repeatability of the results, the entirety of the PTLD sample set was evaluated for peptide C6 and 193–210_A_ reactivity at 2 different timepoints within the study. During these 2 evaluation time points beads were independently coupled, and results were generated months apart that demonstrated comparable results (data not shown). We defined sample positivity as the mean MFI + 6SD, based on previous studies conducted in the Wadsworth Center's clinical laboratories [53, 54]. Index values were calculated using the sample MFI divided by the positivity cutoff for each bead set (ie, each peptide coated bead vs itself) such that an index values of >1.0 indicates positive reactivity for a given bead set.

For validation of the multiplex immunoassays (MIA) on an additional assay platform, enzyme-linked immunosorbent assays (ELISAs) were performed using a subset of the peptides and PTLD serum samples mentioned above. Nunc Maxisorb F96 microtiter plates (Thermo Fisher Scientific) were coated with rOspC or Streptavidin (0.1 μg/well) in PBS (pH 7.4), then incubated overnight at 4°C. The plates were washed 3 times with PBS-Tween 20 (PBS-T; 0.1%, vol/vol) and blocked with goat serum (2%, vol/vol, in PBS-T) for 2 hours at room temperature. For the plates to evaluate the peptides, biotinylated peptides were diluted (1.0 μg/well) and incubated for 1 hour. Plates were washed 3 times before being probed with serum samples (1:50 dilution). Plate-bound antibodies were detected with horseradish peroxidase (HRP)-labeled goat anti-human IgG polyclonal antibodies (SouthernBiotech). The plates were developed with 3,3, 5,5-tetramethylbenzidine (TMB; Kirkegaard & Perry Labs) and analyzed using a SpectraMax iD3 spectrophotometer and SoftMax version 7.1 (Molecular Devices).

### Statistical Analysis

Statistical analyses were performed using R 4.3.0 with R packages readxl and tidyverse [55–57]. MFIs (log_10_) were first subjected to the Shapiro-Wilk's test to assess normality, then either Levene's or Fligner-Killeen tests to compare variances. Mann-Whitney U tests (alpha level of 0.05) with Bonferroni *P* value adjustment were used to determine statistical significance for reactivity determination of OspC subtypes and OspC-derived peptides. R package corrplot [58] was used to generate the Spearman's Rank correlation matrix. R package pheatmap was used to create the hierarchically clustered heatmaps [59]. R packages ggthemes (https://github.com/jrnold/ggthemes), RColorBrewer (https://cran.r-project.org/web/packages/RcolorBrewer/index.html), and ggpubr (https://CRAN.R-project.org/package=ggpubr) were used in formatting.

### Molecular Modeling

PyMol (PyMOL Molecular Graphics System, Version 3.0 Schrödinger, LLC) was used for epitope modeling using OspC PDB ID 1GGQ (strain B31, OspC_A_).

## RESULTS

### Previously Reported Linear B-cell Epitopes on OspC

We sought to validate the reactivity of OspC linear B-cell epitopes, including those already accessioned in the Immune Epitope Database (iedb.org), that have been directly or indirectly implicated in Lyme disease diagnostics and/or *B. burgdorferi* immunity. Eight different epitopes, including a peptide encompassing the largely conserved C-terminus, were chosen for analysis (Table 1). When localized onto the structure of OspC_A_ using PyMol, the epitopes represent ~30% of the surface area of the molecule (Figure 1A). An alignment of the primary amino acid sequences of the 3 OspC types (A, B, K) associated with invasive disease in the Northeast United States revealed polymorphisms in a number of these epitopes (Table 1; Figure 1B) [60, 61]. For this reason, a total of 13 different peptides were generated to encompass the specific amino acid sequences for each epitope within OspC_A_, OspC_B_, and OspC_K_ (Table 1). The peptides were synthesized with a C-terminal biotin tag and coupled to streptavidin-coated microspheres for MIA by Luminex. As a positive control, we also coupled microspheres with the VlsE C6 B31–17 peptide from *B. burgdorferi* strain B31[52].

We had access to 2 *B. burgdorferi* seropositive (Bb^+^) serum sample collections for this study. A “diagnostic” collection consisting of 696 de-identified human serum samples from the Wadsworth Center's Diagnostic Immunology (DI) laboratory previously classified as *B. burgdorferi* seropositive based on Western blot banding profiles (IgM^+^/IgG^-^, IgM^+^/IgG^+^, or IgM^-^/IgG^+^), as described in the Materials and Methods. A second collection provided by the Lyme Disease Biobank at Nuvance Health consisted of 158 serum samples from PTLD patients. These 2 collections were compared to a commercial panel consisting of ~75 serum samples confirmed negative for VlsE C6-17 peptide reactivity. We examined both IgG and IgM (see Supplemental Information) reactivity profiles.

### Contribution of 193–210_A_ to Overall rOspC Antibody Reactivity

**Figure 2.**
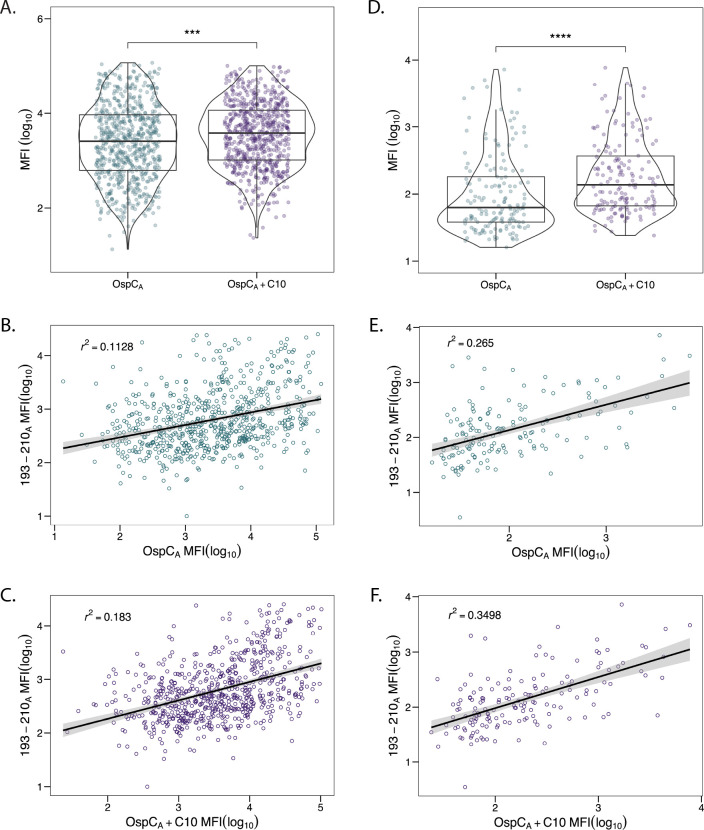
**Correlation between OspC**_A_ **reactivity and C10 region.** Diagnostic (n=686) and PTLD (n=158) samples were subjected to Luminex with OspC_A_, OspC_A_+C10, and 193–210_A_ as described in the Materials and Methods. OspC_A_ MFIs were evaluated against OspC_A_+C10 for diagnostic (A) and PTLD (D) samples. Significance was determined by Mann-Whitney U test. ^*^, *P* < 0.05. To examine the contribution of the C10 region to OspC_A_ reactivity, MFIs for peptide 193–210_A_ were plotted (on the y-axis) versus MFIs from OspC_A_ and OspC_A_+C10 (on the x-axis) for diagnostic samples (B, C) and PTLD samples (E, F). Best fit lines and confidence intervals (level = 0.95) were added to each plot using a linear regression model. Adjusted R-squared (*r*^2^) values were calculated in R.

The C-terminal residues of OspC are an immunodominant linear epitope used in several diagnostic assays [27–31]. However, there is some discrepancy in the literature as to what proportion C-terminus-specific antibodies constitute relative to total OspC antibodies. Some studies have reported that antibodies directed against the terminal 10 residues (C10) account for an overwhelming proportion of the total antibodies to OspC [62]. Others have suggested the opposite [63]. In an effort to resolve this question with our existing collection of Bb^+^ serum samples, we compared antibody reactivity to recombinant OspC_A_ (rOspC_A_) with or without C-terminal residues 202–210 (VVAESPKKP). While MFIs for rOspC_A_ with residues 202–210 were significantly greater than rOspC_A_ without residues 202–210 in both the diagnostic and PTLD serum sample cohorts (Figure 2A-B), the results suggest that reactivity to the C-terminus constitutes only a fraction of the total antibody binding to rOspC_A_.

To address this issue further, we examined serum IgG antibody reactivity to OspC peptide 193–210_A_ (which encompasses the conserved C-terminal amino acids) by Luminex and subjected the resulting MFI values to a linear regression model with MFIs obtained from rOspC_A_ with or without C-terminus. Biotin-labeled OspC peptide 193–210_A_ was coupled to Magplex-avidin microspheres then probed for IgG reactivity with both diagnostic and PTLD serum samples. Linear regression analysis revealed a weak association between OspC peptide 193–210_A_ and rOspC reactivity, irrespective of the presence of the C-terminus (Figure 2B-F). This finding further supports the conclusion that C10-specific antibodies constitute only a small fraction of the total OspC reactivity.

### Serum IgG and IgM Profiling for OspC Linear Epitope Reactivity

**Figure 3.**
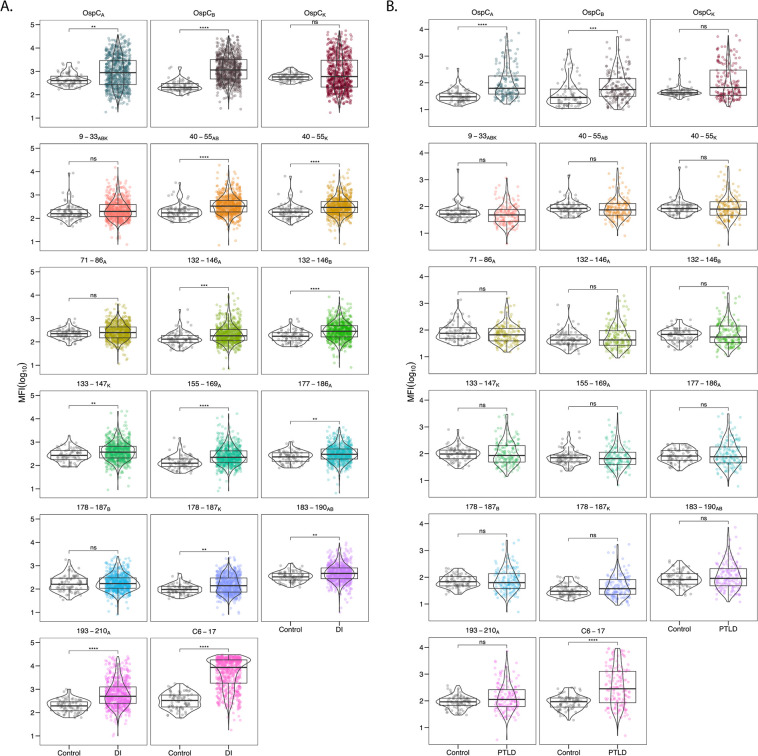
**Serum IgG reactivity of diagnostic and PTLD samples with OspC and OspC-derived peptides.** (A) Diagnostic and (B) PTLD serum samples (diluted 1:100) were subject to MIA using microspheres coated with recombinant dimeric OspC types A, B, and K (top rows), OspC-peptides as described in Table 1, and C6-17 peptide. Panels are labeled by corresponding residue numbers for each OspC type. MFI values were log_10_ transformed and compared to the control sample set. Significance was determined by the Mann-Whitney U-test with Bonferroni *P*-value adjustment (^*^, *P*< 0.05).

The diagnostic and PTLD serum samples were next evaluated in MIA for reactivity with rOspC types A, B, K along with each of the 13 OspC-derived peptides listed in Table 1 in an effort to compare side-by-side relative binding to different peptide (linear) epitopes on OspC. The diagnostic serum IgG samples were significantly reactive with rOspC, with reactivity skewed towards OspC_A_ and OspC_B_ (Figure 3A). The diagnostic samples were also reactive with the 10 of the 13 OspC-derived peptides tested, with the 3 non-reactive peptides being 9–33_ABK_, 71–86_A_, and 178–187_B_ (Figure 3A). The average serum IgG reactivity was highest against OspC peptide 193–210_A_ and VlsE peptide C6-17 whose reactivities were significantly above controls (*P*<1x10^-15^ and *P*< 1x10^-31^, respectively). Analysis of IgM antibodies revealed significant reactivity with rOspC types A and B and peptides 9–33_ABK_ and 178–187_B, K_ but none of the other peptides tested (Supplementary Figure 1). Thus, our results confirm IgG reactivity with previously reported OspC linear epitopes.

**Figure 4.**
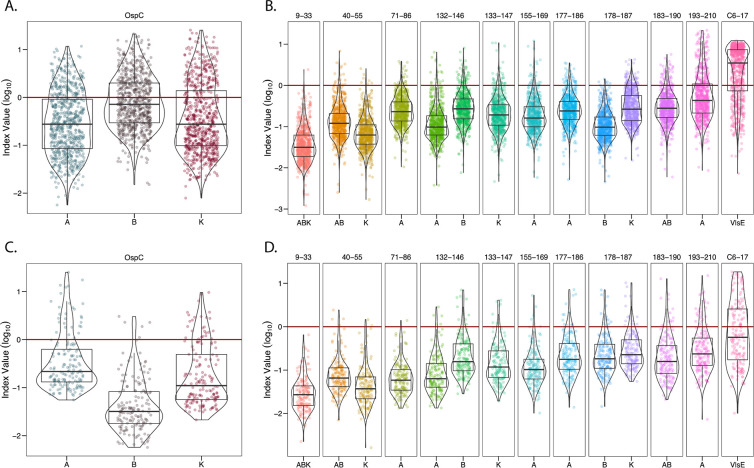
**Indexed (>6SD) IgG reactivity of DI and PTLD serum samples with OspC and OspC-derived peptides.** MFI values for DI (A, B) and PTLD (C, D) serum samples reactive with rOspC (left panels A, C) and OspC- and VlsE-derived peptides (right panels B, D) were indexed using a cutoff of 6SD above mean control reactivity. The resulting values were then log_10_ transformed with each point representing a single sample. OspC subtypes are listed along the x-axis, residues are indicated along the top of the plot, and each antigen is represented by a different color. In each of the graphs, the 6SD cutoff corresponds to a value of 0 on the y-axis and is depicted with horizontal red line. All samples falling above y = 0 were found to be positive for the given antigen and were used to calculate percentage positivity and fold increase as reported in Tables 2 and 3. Box and whisker plots illustrate the upper and lower quartiles as well as the median reactivity to each antigen (thick center line). Sample points that fall above or below the whiskers are considered outliers. Violin plots outline the distribution of data.

From the standpoint of using peptides as diagnostics for Lyme disease, we were particularly interested in peptides that had widespread reactivity within the known Bb^+^ cohorts and high MFI values relative to healthy controls. Garnering such information from previous studies is difficult due to relatively small sample sizes (see references cited in Table 1). To accomplish this, MFI values were subjected to a cutoff value of ≥6SD above the mean of the control MFI, log_10_ transformed, then plotted to reveal both the number of serum samples above that cutoff (% positive) as well as the magnitude (fold increase) of values over the cutoff. We also derived box and whisker plots to illustrate the upper and lower quartiles as well as the median reactivity to each antigen/peptide with superimposed violin plots to outline sample distribution. Using these metrics, reactivity with OspC_A_, OspC_B_, and OspC_K_ ranged from 24% to 42% of the diagnostic samples with the fold increase ranging from 2.8 to 4.7 (Table 2; Figure 4A).

**Table 2.** Significant IgG reactivity (>6SD) to OspC_A/B/K_ and OspC peptides in diagnostic and PTLD serum samples

Diagnostic			PTLD	
OspC or peptide	n = (%)^a^	Fold increase (SD)^b^	n = (%)^a^	Fold increase (SD)^b^
OspC_A_	167 (24.0)	2.8 (1.9)	29 (18.4)	5.5 (5.6)
OspC_B_	287 (41.2)	3.7 (3.4)	5 (3.2)	2.1 (0.6)
OspC_K_	195 (28.0)	4.7 (4.3)	26 (16.5)	2.8 (2.2)
9–33_ABK_	1 (0.1)	2.4 (N/A)	0 (0)	N/A (N/A)
40–55_AB_	17 (2.4)	2.1 (1.4)	4 (2.5)	1.6 (0.5)
40–55_K_	7 (1.0)	1.5 (0.5)	2 (1.3)	1.3 (0.2)
71–86_A_	28 (4.0)	1.7 (0.8)	1 (0.6)	1.4 (NA)
132–146_A_	18 (2.6)	2.4 (1.5)	5 (3.2)	1.7 (0.6)
132–146_B_	48 (6.9)	2.0 (1.3)	11 (7.0)	3.0 (1.9)
133–147_K_	36 (5.2)	2.4 (2.6)	8 (5.1)	2.5 (1.2)
155–169_A_	30 (4.3)	2.5 (2.5)	5 (3.2)	2.5 (1.5)
177–186_A_	38 (5.5)	1.6 (0.7)	13 (8.2)	2.6 (2.0)
178–187_K_	70 (10.1)	1.5 (0.7)	15 (9.5)	3.0 (2.8)
178–187_B_	7 (1.0)	1.2 (0.1)	12 (7.6)	2.7 (1.9)
183–190_AB_^c^	53 (7.6)	1.9 (1.0)	14 (8.9)	3.0 (3.1)
193–210_A_ (C10)	185 (26.6)	4.4 (4.5)	22 (14.0)	3.3 (3.0)
VlsE C6-17	496 (71.3)	5.8 (2.9)	64 (40.5)	5.6 (5.0)

^a^, The number of samples (n =) and percent reactivity (%) to each antigen in the Diagnostic (n=696) and PTLD (n=158) sample sets were calculated using a cutoff of >6SD above the mean of control samples;

^b^, The fold increase (magnitude) of positive sample antibody reactivity over controls such that an index value of 1 is 6SD above the control mean. For example, 2.8 corresponds to ~16SD greater than the mean control.

^c^, OspC_B_ residues 184–191.

In terms of peptides, VlsE C6-17 was the most reactive (71% positive; fold increase 5.8), followed by 193–210_A_ (26% positive; fold increase 4.4) (Table 2; Figure 4B). The percentage of samples >6SD for the other 12 peptides ranged from 0.1% to 10% with the fold increase between 1.2 and 2.5 (Table 2; Figure 4B). Lowering the cutoff threshold to >3SD resulted in higher overall peptide reactivities, as expected, but the pattern remained the same as >6SD (Supplementary Table 1). Thus, within a given cohort of *B. burgdorferi* seropositive samples, high serum IgG reactivity to OspC-derived peptides (apart from 193–210_A_) is limited to a small subset of individuals, which has important consequences for use of such peptides in diagnostic applications. In terms of IgM reactivity, the trends were similar to IgG except that the most pronounced antibody interactions were with peptides 177–186_B, K_ followed by 9–33 _ABK_ (Supplementary Table 2). The differential peptide reactivity profiles between IgG and IgM may have diagnostic utility.

The same analysis was performed with the panel of PTLD samples. The PTLD samples were highly reactive with OspC_A_ and OspC_B_ with 16% and 18% of the samples having MFIs >6SD cutoff and fold increases between 2.8 and 5 (Table 2; Figures 3B, 4C). In the case of OspC_K_, only 3.2% were above the cutoff with a fold increase of 2.1 (Table 2; Figure 4C). In terms of linear epitopes, reactivity significantly above controls was limited to VlsE C6-17 (40% positive; fold increase 5.6) (Table 2: Figure 3B; Figure 4D). Only a small fraction of the PTLD serum samples were found to be positive for the 13 OspC peptides (range 0% to 14%) and the MFIs did not achieve statistical significance (Table 2; Figure 3B). PTLD samples were plotted to visualize OspC and peptide reactivities relative to the cutoff (Figure 4C-D). There is notably less reactivity to both 193–210_A_ and C6-17 in the PTLD cohort in comparison to the diagnostic serum set.

**Figure 5.**
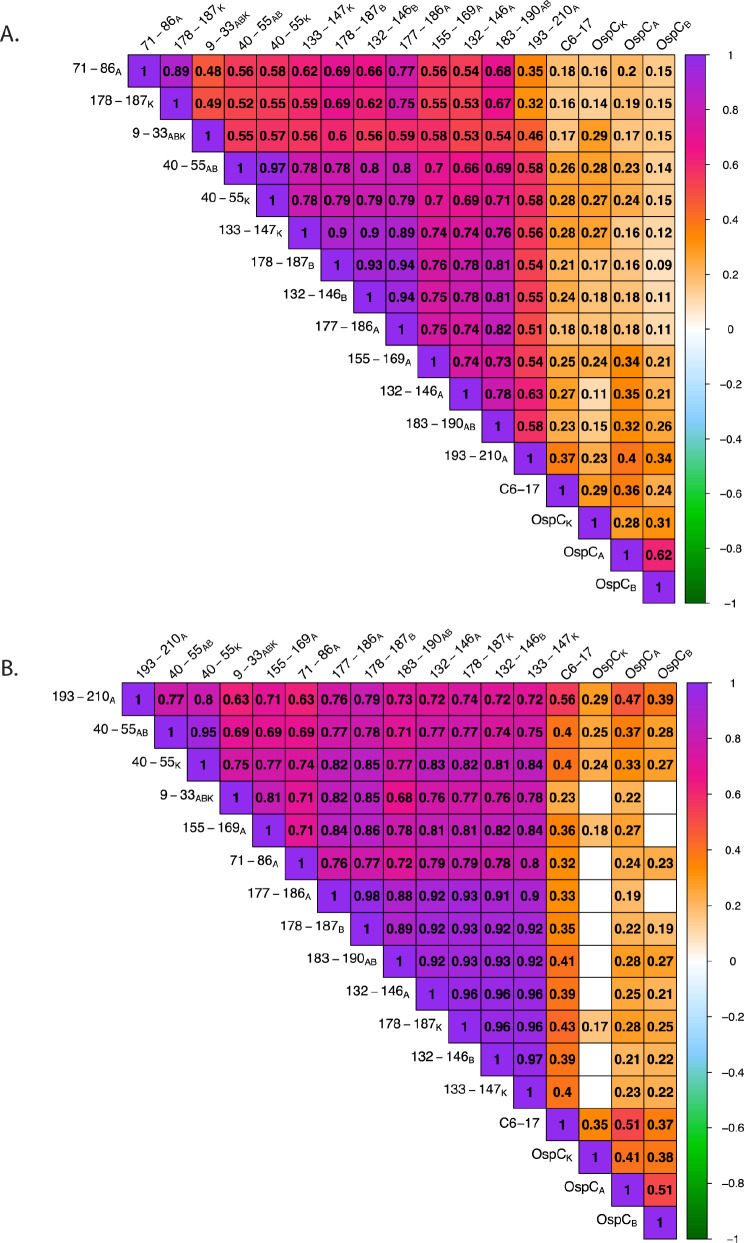
**Correlation matrices of rOspC and OspC-derived peptide reactivity in diagnostic and PTLD serum.** Diagnostic (A) and PTLD (B) serum sample MFIs (indexed and log_10_ transformed) were subjected to Spearman's Rank correlation. Only significant correlation coefficients (r_s_) are displayed (confidence level = 0.95). The degree and direction of correlation is indicated by the color scale. Interpretations of the strength of each correlation was conducted as such: >0.7 indicates a strong correlation, 0.5 – 0.7 is moderate, 0.3 – 0.5 is weak, and 0.0 – 0.3 is negligible.

We postulated that antibody reactivity with the OspC-derived peptides would correlate with overall OspC antibody levels in any given sample, assuming that the 2 antibody populations track with each other and serve as redundant indicators of a previous *B. burgdorferi* infection. To investigate these relationships, we generated correlation matrices (Spearman's Rank) assessing the correlation between OspC and each of the different OspC-derived peptides, including 193–210_A_, and VlsE C6-17 (Figure 5). While all correlations were determined to be positive, peptide 193–210_A_ reactivity was only weakly correlated with OspC_A_, OspC_B_, or OspC_K_ reactivity (r_s_ = 0.2–0.4) in the diagnostic samples (Figure 5A). Rather, 193–210_A_ reactivity was moderately to strongly correlated with other OspC-derived peptides, including 132–146_A_ (r_s_ = 0.63), 183–190_AB_ (r_s_ = 0.58), and 40–55_ABK_ (r_s_ = 0.58). Indeed, inter-peptide correlations were generally strong across the board (eg, 132–146_B_ versus 177–186_A_, r_s_ > 0.9). A similar pattern was observed in the PTLD samples: OspC-derived peptide reactivities were strongly correlated with each other (r_s_ = 0.63–0.98), but weakly correlated with OspC_A_, OspC_B_, and OspC_K_ (r_s_ = 0.2–0.4) (Figure 5B). In the case of VlsE C6-17, reactivity was weakly correlated with OspC and OspC-derived peptides in both the diagnostic and PTLD samples, except for moderate correlativity with 193–210_A_ (C10) in the PTLD cohort (r_s_ = 0.56). These results reveal a significant degree of discordance (asynchrony) among circulating antibody levels against OspC, OspC-derived peptides, and the VlsE C6-17 peptide in both diagnostic and PTLD cohorts.

**Figure 6.**
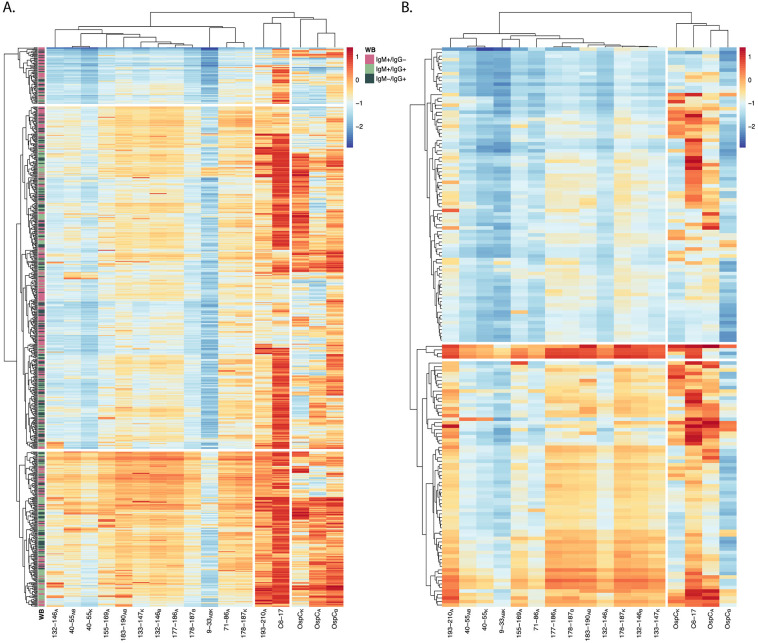
**Hierarchical clustering of rOspC and OspC-derived reactivity in diagnostic and PTLD serum.** Heatmaps were generated using diagnostic (A) and PTLD (B) serum sample MFIs (indexed and log_10_ transformed). Rows represent individual samples and columns represent the panel of antigens. The color scales of A and B are identical to one another (allowing for direct comparison), with 0 representing the positivity cutoff (as seen in Figure 4). Annotation of the diagnostic samples (A) with Western blot profile data (previously described in the Materials and Methods) is designated by the legend. The columns were subjected to hierarchical clustering by correlation and the rows were clustered by Euclidean distance to better visualize patterns in reactivity profiles.

The absence of a strong correlation between antibody levels to OspC and OspC-derived peptides led us to subject the diagnostic and PTLD datasets to hierarchical clustering (HC) as a means to identify possible relationships in immunoreactivity profiles. In the case of the diagnostic samples, the horizontal (top) dendrogram revealed 3 distinct branches consisting of (1) OspC_A_, OspC_B_, and OspC_K_, (2) C6-17 and 193–210_A_ (C10), and (3) the other OspC-derived peptides (Figure 6A). This patterning reinforces the idea that the circulating antibody profiles to OspC and its immunodominant (ie, 193–210_A_) and subdominant linear epitopes are asynchronous and not directly correlated with each other. Moreover, there was no obvious clustering on the vertical dendrogram to indicate a relationship between antibody reactivity profiles and seropositivity categories determined in the 2-tiered Lyme disease diagnostic test (ie, IgM^+/-^, IgG^+/-^).

The HC plot of the PTLD serum samples was distinct from the diagnostic samples in that there were just 2 major horizontal branches not 3: one encompassing OspC_ABK_ and VlsE C6-17, and the other containing the OspC-derived peptides, including 193–210_A_ (Figure 6B). This same clustering was observed when a subset of PTLD samples were evaluated by ELISA for peptide and OspC reactivity, demonstrating that the results are valid across assay platforms (Supplementary Figure 2). On the vertical dendrogram, there were 2 branches: the top branch corresponding to low peptide reactivity with or without concomitant C6-17 and OspC reactivities and the bottom branch corresponding to high peptide reactivity that were more frequently than not associated with high C6-17 reactivity. Within the bottom branch, there was a notable subbranch corresponding to 4 samples with strikingly high reactivity with 193–210_A_, C6-17, and most of the OspC-derived peptides (including 9–33_ABK_). We would tentatively classify these handful of samples as “polyreactive” [64]. In summary, HC analysis of both the diagnostic and PTLD cohorts confirms the asynchronous nature of serum antibody profiles associated with rOspC and OspC-derived peptides that may, in fact, reflect the intrinsically complicated immune response to *B. burgdorferi* infection in the first place.

## DISCUSSION

Defining the breadth and the frequency of linear B-cell epitope reactivity on OspC has important implications for both Lyme disease diagnostics and vaccines. In this report, a panel of peptides representing 8 previously reported and predicted linear epitopes on OspC types A, B, and K, were examined by multiplex immunoassay for serum IgG reactivity in diagnostic (n~700) and PTLD (n~150) *B. burgdorferi* seropositive sample sets. The results revealed that 6 of the 8 peptides are indeed antibody targets even though high reactivity to any given peptide was confined to only a fraction (2% to 10%) of the total. The exception being peptide 193–210_A_, which was 26% positive (4.4-fold increase value) in the diagnostic sample set and 14% positive (3.3-fold increase value) in the PLTDs sample set. Interestingly, in the diagnostic sample set, 193–210_A_ antibody titers did not correlate with either reactivity to OspC itself or any of the other OspC-derived peptides. In the PTLD sample set, 193–210_A_ antibody levels did correlate with the other OspC-derived peptides but were distinct from OspC. These results have clinical implications, as they demonstrate that the human B-cell response to linear epitopes on OspC is both variable and asynchronous.

The 193–210_A_ peptide includes the stretch of ~10 highly conserved amino acids at C-terminus of OspC, first recognized as an immunodominant epitope in neuroborreliosis patients [62]. Since then, the so-called C10 peptide and derivatives have been integrated into numerous diagnostic assays, including the Zeus VlseE1/C10 ELISA [26, 28, 29]. Dolange and colleagues recently proposed the use of high affinity C10-specific monoclonal antibodies as tools to detect circulating levels of *B. burgdorferi* antigens [65]. While there is little debate as to whether C10 constitutes a human B-cell epitope, the issue of whether antibodies to C10 contribute to complement-dependent, antibody-mediated killing of *B. burgdorferi* (“borreliacidal activity”) remains contentious. Lovrich and colleagues reported that OspC-specific borreliacidal activity (in a handful of human serum samples) was almost entirely directed against C10 [33, 34], whereas Izac and colleagues argued that, in rodents, canines, and non-human primates, C10-specific antibodies contribute little if any borreliacidal activity [66].

Although our results do not resolve this issue per se, they do raise questions about the underlying B-cell biology associated with antibodies to C10 and OspC. The fact that C10 and OspC antibody titers did not correlate with each other in the 2 serum sample sets examined suggests differences in the kinetics of antibody induction, circulating half-life, and/or plasma cell persistence. Others have suggested, at least in the case of VlsE, that there is a distinct progression of epitope reactivity over the course of *B. burgdorferi* infection with readily accessible epitopes targeted first and more obstructed, membrane proximal epitopes following [67]. Resolving detailed questions about the nature of the human B-cell response to OspC is becoming more feasible as single cell V_H_ and V_L_ repertoires are catalogued longitudinally over the course of Lyme disease [12, 68].

B-cell epitope prediction software like Discotope identifies residues 133–147 as having a high immunogenic propensity [69]. That prediction was borne out in our study, as evidenced by statistically significant reactivity of serum samples with the 3 peptides corresponding to residues 132–146_AB_ and 133–147_K_. Others have reported similar findings in mice and in a few human samples. Namely, Earnhart recognized residues 136–150 (which they refer to as “loop 5”) as a target of antibodies in *B. burgdorferi*-infected mice [35]. In a follow-up study, the same investigators identified 2 serum samples from patients with early Lyme disease that reacted with peptides spanning 136–144 [37]. A subsequent report by Arnaboldi and colleagues did not detect binding to 3 different 15-mer OspC_K_-derived peptides spanning residues 131–155, possibly because their sample size was limited [28]. In our collection of almost 700 diagnostic samples, the frequency of peptide positivity was admittedly relatively low (3% to 7%). Nonetheless, antibodies targeting this epitopic region may be consequential in terms of promoting spirochete clearance [35]. For example, the potent borreliacidal mouse monoclonal antibody 16.22 recognizes OspC_A_ residues 133–147 with the key contact points postulated to be residues 139–141 (KXK motif) [36]. Thus, a more detailed examination of B-cell epitopes within this region of OspC in human patients with Lyme disease is warranted.

It has been postulated that serum antibody profiling at the antigen and possibly even epitope level may be able to resolve different stages of Lyme disease (eg, acute, acute resolved, PTLD) [10, 67]. While the antibody profiling in our study was limited to serum IgG and IgM reactivity to OspC, OspC-derived peptides, and VlsE C6-17, the results revealed distinct patterns between the diagnostic and PTLD sample sets when subjected to HC. Most notable was the classification of the C10 peptide: in the diagnostic sample set C10 clustered with C6-17, whereas in the PTLD samples it clustered with the other OspC-derived peptides. Thus, it is tempting to speculate that the ratio of C10 to C6-17 serum IgG reactivity may have some utility in defining disease progression. By the same token, as virtually all samples tested were positive for either C10 and/or C6-17, establishing a combined C10 + C6-17 threshold value might serve to alleviate false positives in Lyme disease diagnostics based solely on serology [70, 71]. That said, we recognize that any claims to this effect are premature, considering that the serum samples employed in our study are not associated with any clinical information beyond seropositivity. Moreover, we lack a “post-Lyme healthy” cohort that Chandra and colleagues so effectively used in their study to compare with their PTLD cohort [10].

One interesting grouping that emerged from the PTLD cohort was a cluster of 4 individuals who demonstrated pan-reactivity with the OspC-derived peptides and VlsE C6-17 peptide. In follow-up analysis, those same 4 samples in question also reacted with other *B. burgdorferi*-derived peptides, as well as control non-*B. burgdorferi*-derived peptides, but not necessarily with the corresponding recombinant proteins (G. Freeman-Gallant, unpublished results). Thus, the serum antibodies from those particular individuals seemingly have a propensity for peptide antigens, possibly due to a particular bias in germline chain usage and corresponding paratopes conducive to accommodating peptides [72]. Whether or not there is a germline bias in antibodies associated with PTLD is worthy of investigation, especially in light of findings related to COVID-19. Moreover, the availability of bulk plasmablast BCR sequences, as well as single-cell paired BCR sequences from patients with Lyme disease opens the door to detailed antibody reactivity profiles and links to disease manifestations [73]. We are also exploring the possibility that these particlar samples may have profiles consistent with autoantibodies [74] or have characteristics associated with polyreactivity that might promote ligation to spirochete surface antigens [75, 76].

There are 2 notable shortcomings associated with this study that need to be underscored. First, as alluded to above, is our reliance on remnant serum samples with limited clinical attributes associated with them, rather than using samples procured as part of a prospective study of patients with Lyme disease in which serum profiles might be linked to stages of disease progression and resolution [67]. Moreover, the serum samples are biased in that they were already deemed positive in the 2-tiered Lyme disease assay, which includes VlsE and/or OspC antigens or epitopes, and from a limited geographical area (Northeast United States). Second are the intrinsic limitations associated with peptide-based epitope reactivity profiling, as detailed in a recent review [77]. Specifically, polypeptides, whether in solution or bound to a matrix, do not necessarily assume a “native” secondary structure. As a result, low or no antibody reactivity to a given peptide does not necessarily indicate that the corresponding region of the native antigen is not immunogenic. Nonetheless, we employed a variety of methods to validate our peptide epitopes wherever possible, including (1) using peptides previously reported in the literature as being reactive serum samples from patients with Lyme disease as detailed in Table 1; (2) using monoclonal antibodies (MAb) to validate the peptide epitopes [65, 78]; (3) validating epitope specificity by alanine scanning [78]; (4) coupling peptides to Luminex beads via C-terminal GGGSK-biotin linker rather than direct chemical or passive conjugation; and (5) employing commercial Lyme disease seronegative and seropositive samples as intra-assay and bead coupling confirmation controls, as described in the Materials and Methods. Despite these limitations, our study involving the profiling of >800 unique Bb+ serum samples provides important insights into epitope usage across the surface of OspC that will have implications for diagnostic and vaccine implementation.

## Footnotes

1 It has been proposed that the Lyme disease spirochetes be reclassified as the genus *Borreliella*, although this proposal has not yet been widely accepted within the community. See: Adeolu M, Gupta RS. A phylogenomic and molecular marker-based proposal for the division of the genus Borrelia into 2 genera: the emended genus Borrelia containing only the members of the relapsing fever Borrelia, and the genus Borreliella gen. nov. containing the members of the Lyme disease Borrelia (Borrelia burgdorferi sensu lato complex). Antonie Van Leeuwenhoek. 2014 Jun;105(6):1049-1072. doi: 10.1007/s10482-014-0164-x. Epub 2014 Apr 18. PMID: 24744012. and Margos G, Marosevic D, Cutler S, Derdakova M, Diuk-Wasser M, Emler S, Fish D, Gray J, Hunfeldt KP, Jaulhac B, Kahl O, Kovalev S, Kraiczy P, Lane RS, Lienhard R, Lindgren PE, Ogden N, Ornstein K, Rupprecht T, Schwartz I, Sing A, Straubinger RK, Strle F, Voordouw M, Rizzoli A, Stevenson B, Fingerle V. There is inadequate evidence to support the division of the genus Borrelia. *Int J Syst Evol Microbiol*. 2017 Apr;67(4):1081-1084. doi: 10.1099/ijsem.0.001717. Epub 2017 May 5. Erratum in: *Int J Syst Evol Microbiol*. 2017 Jun;67(6):2073. doi: 10.1099/ijsem.0.002100. Hunfeldt, K-P (corrected to Hunfeld, K-P). PMID: 27930271.

## SUPPLEMENTARY DATA

Supplementary materials are available at the *Pathogens and Immunity* website. Supplementary data may be provided by the authors to benefit the reader. Supplementary data are not copyedited and are the sole responsibility of the authors. Questions or comments related to supplementary materials should be addressed to the corresponding author.
