# Supplementary material for: A Refined Human Linear B Cell Epitope Map of Outer Surface Protein C (OspC) From the Lyme Disease Spirochete, Borrelia Burgdorferi
Source: Pathog Immun. 2025 Feb 14;10(1):159–86. doi: 10.20411/pai.v10i1.756 (PMC11867186; doi:10.20411/pai.v10i1.756)
Supplement: Supplementary Figures and Tables [file pai-10-159-s01.pdf]

PATHOGENS AND IMMUNITY
In Association with Case Western Reserve University and The Richard J. Fasenmyer Foundation

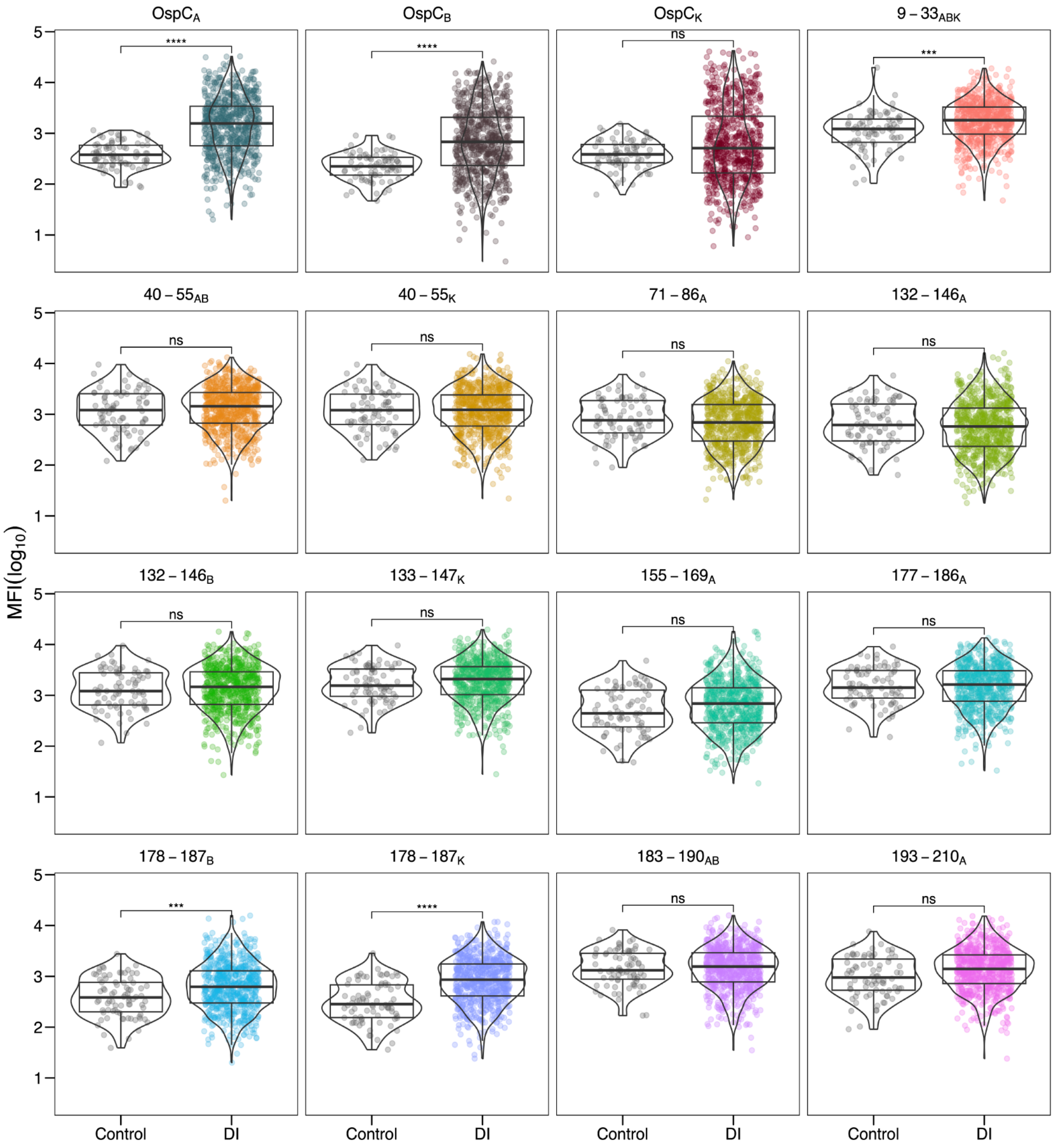

Supplementary Figure 1. Serum IgM reactivity of diagnostic samples with OspC and OspC-derived peptides. DI serum samples (diluted 1:100) were subject to MIA using microspheres coated with recombinant dimeric OspC types A, B and K and OspC-peptides as described in Table 1. Panels are labeled by corresponding residue numbers for each OspC type. MFI values were log10 transformed and compared to the control sample set. Significance was determined by the Mann-Whitney U-test and Student t-test depending on data distribution (*, $P < 0.05$).

Freeman-Gallant G, McCarthy K, Yates J, Kulas K, Rudolph MJ, Vance DJ, Mantis NJ. A Refined Human Linear B Cell Epitope Map of Outer Surface Protein C (OspC) From the Lyme Disease Spirochete, *Borrelia burgdorferi*. *Pathogens and Immunity*. 2025;10(1):159–186. doi: 10.20411/pai.v10i1.756

PATHOGENS AND IMMUNITY
IN ASSOCIATION WITH CASE WESTERN RESERVE UNIVERSITY AND THE RICHARD J. FASENMYER FOUNDATION

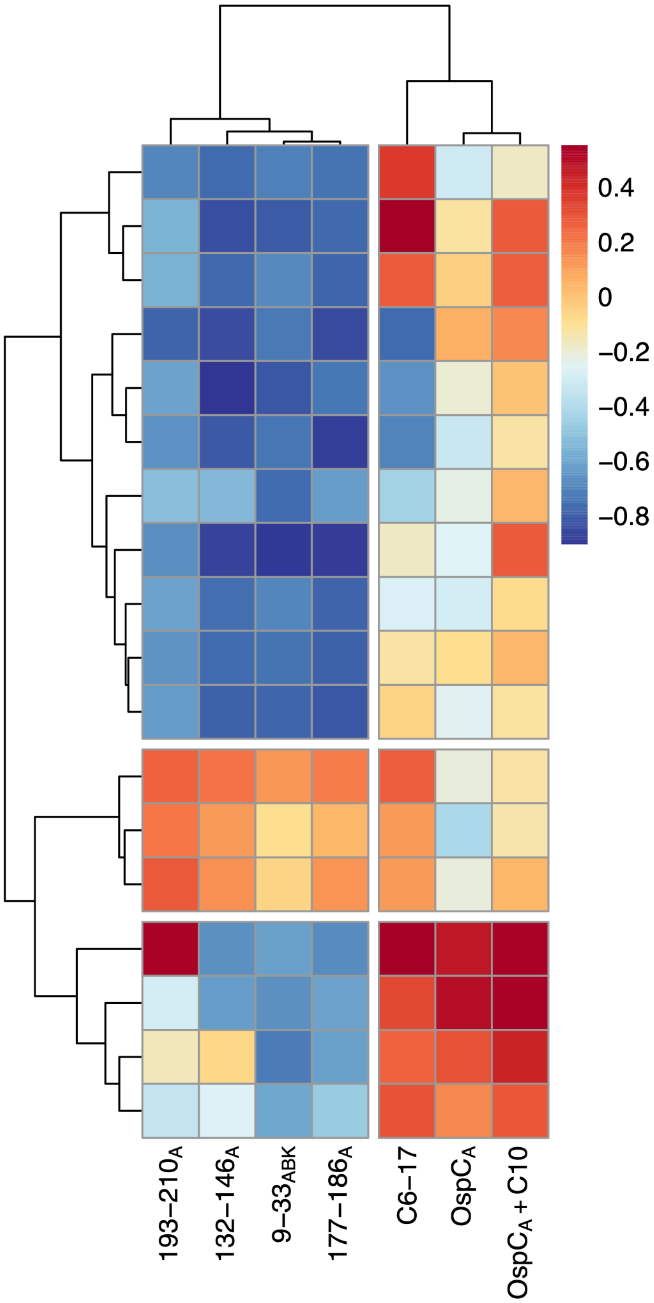

Supplementary Figure 2. Hierarchical clustering of rOspC and OspC-derived reactivity in diagnostic and PTLD serum by ELISA. PTLD serum samples (diluted 1:50; n=18) were subject to ELISA using microtiter plates coated with recombinant dimeric OspCA and a subset of the OspC-peptides described in Table 1. Heatmaps were generated using PTLD serum sample log10 transformed OD results. Rows represent individual samples and columns represent the panel of antigens. The columns were subjected to hierarchical clustering by correlation and the rows were clustered by Euclidean distance to better visualize patterns in reactivity profiles.

Freeman-Gallant G, McCarthy K, Yates J, Kulas K, Rudolph MJ, Vance DJ, Mantis NJ. A Refined Human Linear B Cell Epitope Map of Outer Surface Protein C (OspC) From the Lyme Disease Spirochete, *Borrelia burgdorferi*. *Pathogens and Immunity*. 2025;10(1):159–186. doi: 10.20411/pai.v10i1.756

PATHOGENS AND IMMUNITY
In Association with Case Western Reserve University and The Richard J. Fasenmyer Foundation

Supplementary Table 1. IgG reactivity (>3SD) to $OspC_{A/B/K}$ and OspC peptides in diagnostic and PTLD serum samples

	Diagnostic		PTLD	
**OspC / peptide**	**n = (%)[a]**	**Fold increase (SD)[b]**	**n = (%)[a]**	**Fold increase (SD)[b]**
$OspC_A$	240 (34.5)	3.7 (3.2)	43 (27.2)	7.0 (9.0)
$OspC_B$	388 (55.7)	5.0 (5.4)	6 (3.8)	3.6 (1.3)
$OspC_K$	243 (34.9)	6.1 (6.4)	38 (24.1)	3.9 (3.7)
$9\text{-}33_{ABK}$	7 (1.0)	1.8 (1.2)	1 (0.6)	1.2 (N/A)
$40\text{-}55_{AB}$	45 (6.5)	2.3 (2.0)	7 (4.4)	2.3 (1.1)
$40\text{-}55_K$	16 (2.3)	1.9 (1.0)	5 (3.2)	1.8 (0.6)
$71\text{-}86_A$	92 (13.2)	1.7 (1.1)	5 (3.2)	1.5 (0.5)
$132\text{-}146_A$	46 (6.6)	2.5 (2.2)	9 (5.7)	2.4 (1.3)
$132\text{-}146_B$	118 (17.0)	2.1 (1.8)	21 (13.3)	3.2 (2.9)
$133\text{-}147_K$	75 (10.8)	2.6 (3.3)	15 (9.5)	2.9 (2.1)
$155\text{-}169_A$	75 (10.8)	2.6 (3.3)	10 (6.3)	2.8 (2.4)
$177\text{-}186_A$	82 (11.8)	1.9 (1.0)	28 (17.7)	2.7 (2.7)
$178\text{-}187_K$	164 (23.6)	1.8 (1.0)	30 (19.0)	3.1 (3.7)
$178\text{-}187_B$	24 (3.4)	1.5 (0.4)	25 (15.8)	2.7 (2.7)
$183\text{-}190_{AB}$[c]	110 (15.8)	2.1 (1.4)	22 (13.9)	3.7 (4.5)
$193\text{-}210_A$ (C10)	272 (39.1)	5.4 (6.8)	36 (22.8)	3.8 (4.3)
VlsE C6-17	537 (77.2)	9.2 (5.3)	78 (49.4)	7.8 (8.1)

[a], The number of samples (n =) and percent reactivity ("%") to each antigen in the diagnostic (n=696) and PTLD (n=158) sample sets were calculated using a cutoff of >3SD above the mean of controls samples; [b], The fold increase of positive sample antibody reactivity over controls such than an index value of 1 is 3SD above the control mean, while 3.7 corresponds to ~11SD greater than the mean control. [c], $OspC_B$ residues 184-191

Freeman-Gallant G, McCarthy K, Yates J, Kulas K, Rudolph MJ, Vance DJ, Mantis NJ. A Refined Human Linear B Cell Epitope Map of Outer Surface Protein C (OspC) From the Lyme Disease Spirochete, *Borrelia burgdorferi*. *Pathogens and Immunity*. 2025;10(1):159–186. doi: 10.20411/pai.v10i1.756

PATHOGENS AND IMMUNITY
IN ASSOCIATION WITH CASE WESTERN RESERVE UNIVERSITY AND THE RICHARD J. FASENMYER FOUNDATION

Supplementary Table 2. Serum IgM reactivity greater than 3SD and 6SD to $OspC_{A/B/K}$ and OspC peptides in diagnostic serum samples

	3SD	6SD
**OspC / peptide**	**n = (%)[a]**	**n = (%)[a]**
$OspC_A$	398 (57.2)	306 (44.0)
$OspC_B$	309 (44.4)	234 (33.6)
$OspC_K$	219 (31.5)	172 (24.7)
$9\text{-}33_{ABK}$	22 (3.2)	5 (0.7)
$40\text{-}55_{AB}$	25 (3.6)	1 (0.1)
$40\text{-}55_K$	18 (2.6)	5 (0.7)
$71\text{-}86_A$	24 (3.4)	4 (0.6)
$132\text{-}146_A$	32 (4.6)	9 (1.3)
$132\text{-}146_B$	32 (4.6)	4 (0.6)
$133\text{-}147_K$	38 (5.5)	6 (0.9)
$155\text{-}169_A$	57 (8.2)	19 (2.7)
$177\text{-}186_A$	27 (3.9)	1 (0.1)
$178\text{-}187_K$	170 (24.4)	61 (8.8)
$178\text{-}187_B$	100 (14.4)	40 (5.7)
$183\text{-}190_{AB}$[b]	32 (4.6)	5 (0.7)
$193\text{-}210_A$ (C10)	57 (8.2)	14 (2.0)

[a], The number of samples (n =) and percent reactivity ("%") to each antigen in the diagnostic (n=696) sample set were calculated using cutoffs of >3SD and >6SD above the mean of controls samples; [b], Residues 184-191 in Type B.

Freeman-Gallant G, McCarthy K, Yates J, Kulas K, Rudolph MJ, Vance DJ, Mantis NJ. A Refined Human Linear B Cell Epitope Map of Outer Surface Protein C (OspC) From the Lyme Disease Spirochete, *Borrelia burgdorferi*. *Pathogens and Immunity*. 2025;10(1):159–186. doi: 10.20411/pai.v10i1.756
